# Eliminating Both Canonical and Short-Patch Mismatch Repair in *Drosophila melanogaster* Suggests a New Meiotic Recombination Model

**DOI:** 10.1371/journal.pgen.1004583

**Published:** 2014-09-04

**Authors:** K. Nicole Crown, Susan McMahan, Jeff Sekelsky

**Affiliations:** 1 Department of Biology, University of North Carolina, Chapel Hill, Chapel Hill, North Carolina, United States of America; 2 Integrative Program for Biological and Genome Sciences, University of North Carolina, Chapel Hill, Chapel Hill, North Carolina, United States of America; 3 Lineberger Comprehensive Cancer Center, University of North Carolina, Chapel Hill, Chapel Hill, North Carolina, United States of America; Columbia University, United States of America

## Abstract

In most meiotic systems, recombination is essential to form connections between homologs that ensure their accurate segregation from one another. Meiotic recombination is initiated by DNA double-strand breaks that are repaired using the homologous chromosome as a template. Studies of recombination in budding yeast have led to a model in which most early repair intermediates are disassembled to produce noncrossovers. Selected repair events are stabilized so they can proceed to form double-Holliday junction (dHJ) intermediates, which are subsequently resolved into crossovers. This model is supported in yeast by physical isolation of recombination intermediates, but the extent to which it pertains to animals is unknown. We sought to test this model in *Drosophila melanogaster* by analyzing patterns of heteroduplex DNA (hDNA) in recombination products. Previous attempts to do this have relied on knocking out the canonical mismatch repair (MMR) pathway, but in both yeast and *Drosophila* the resulting recombination products are complex and difficult to interpret. We show that, in *Drosophila*, this complexity results from a secondary, short-patch MMR pathway that requires nucleotide excision repair. Knocking out both canonical and short-patch MMR reveals hDNA patterns that reveal that many noncrossovers arise after both ends of the break have engaged with the homolog. Patterns of hDNA in crossovers could be explained by biased resolution of a dHJ; however, considering the noncrossover and crossover results together suggests a model in which a two-end engagement intermediate with unligated HJs can be disassembled by a helicase to a produce noncrossover or nicked by a nuclease to produce a crossover. While some aspects of this model are similar to the model from budding yeast, production of both noncrossovers and crossovers from a single, late intermediate is a fundamental difference that has important implications for crossover control.

## Author Summary

During meiosis, breaks are introduced into the DNA, then repaired to give either crossovers between homologous chromosomes (these help to ensure correct segregation of these chromosomes from one another), or non-crossover products. Meiotic break repair mechanisms have been best studied in budding yeast, leading to detailed molecular models. Technical limitations have prevented directly testing these models in multi-cellular organisms. One approach that has been tried is to map segments of DNA that are mismatched, since different models predict different arrangements. Mismatches are usually repaired quickly, so analyzing these patterns requires eliminating mismatch repair processes. Although others have knocked out the primary mismatch repair system, we have now, for the first time in an animal, identified the secondary repair pathway and eliminated it and the primary pathway simultaneously. We then analyzed mismatches produced during meiosis. Though the results can be fit to the most popular current model from yeast, if some modifications are made, we also consider a simpler model that incorporates elements of the current model and of earlier models.

## Introduction

**Figure 1:**
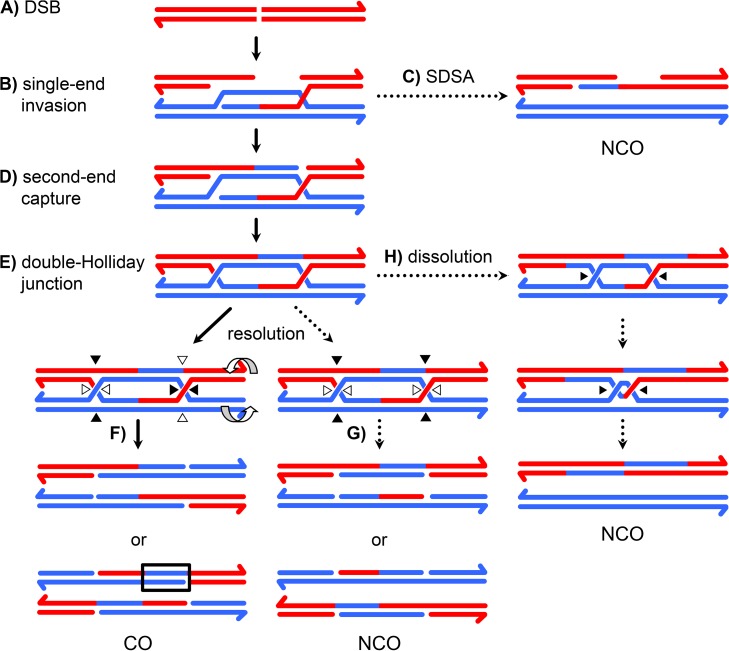
A current model of meiotic recombination. (**A**) A double-strand break (DSB) is processed to form 3′ single strand overhangs. (**B**) One of the single strands invades the homologous chromosome, forming a single-end invasion intermediate. (**C**) After synthesis, this intermediate may be disassembled, allowing the newly synthesized DNA to anneal to the other resected end. This process, called synthesis dependent strand annealing (SDSA), generates NCOs only. This NCO is drawn with hDNA intact, but the nicks in the product would likely stimulate mismatch repair, possibly leading to a single tract of gene conversion to one side of the DSB. (**D**) In the absence of SDSA, the second end of the DSB may be captured by annealing to the displaced strand of the D-loop, priming synthesis. (**E**) This structure is ligated to form a double Holliday junction (dHJ). The HJs are cleaved in a process called resolution. (**F**) Cleaving different strands at each junction (left) results in a CO. One way of doing this (open arrowheads) results in products with a single hDNA tract; the other orientation (black arrowheads) gives products with MMR-independent gene conversion (outlined in black) adjacent to the tract of hDNA. (**G**) Cutting the same two strands at both junctions (right) results in a NCO. Both orientations give one product with a single tract of hDNA and one with hDNA adjacent to a gene conversion tract. Resolution, like SDSA, leaves nicks in the final products that are thought to direct mismatch repair. (**H**) dHJs may be dissolved by the combined activities of a helicase and topoisomerase, resulting in a NCO with *trans* hDNA and lacking nicks.

Meiotic recombination is initiated by a DSB on one chromatid followed by repair using the homologous chromosome as a template, resulting in crossover (CO) or noncrossover (NCO) products [1]. In the predominant model of repair, NCOs are produced when an early intermediate – a D-loop extended by synthesis using a homologous template – is disassembled by a helicase (Figure 1C), whereas COs are produced when a late intermediate – the double-Holliday junction (dHJ) – is cleaved by a resolvase (Figure 1F). Crossover control, the ill-defined mechanisms that determine the number and distribution of crossovers, is thought to act prior to the bifurcation of CO and NCO pathways [2].

This model has been derived largely from studies in *Saccharomyces cerevisiae*, with strong support coming from the physical isolation of molecules with the properties expected of the key intermediates [3], [4]. Because many key meiotic recombination proteins are conserved, it is thought that this model is also applicable to plants and animals; however, it has not been possible to isolate recombination intermediates in model metazoans to test this assumption. Here, we take a molecular genetic approach to analyzing recombination intermediates to determine what structures give rise to COs and NCOs in a model metazoan, *Drosophila melanogaster*.

Recombination involves formation of heteroduplex DNA (hDNA), regions in which the two strands of a duplex come from different parental DNA molecules (Figure 1). Sequence differences between the parental chromosomes result in base-base mismatches and insertion/deletion (indel) loops in hDNA and can be used as markers to map hDNA tracts. Different recombination models predict different arrangements of hDNA (*e.g.*, Figure 1C vs 1H). In the budding yeast model, NCOs arise from synthesis-dependent strand annealing (SDSA), with limited, if any, contribution from dHJ resolution or dissolution. SDSA predicts a *cis* configuration of hDNA, with all of the markers from the donor on one strand of the product (Figure 1C). In contrast, dHJ dissolution predicts *trans* hDNA, with markers on different strands on opposite sides of the DSB (Figure 1H). Crossovers are thought to come from resolution of dHJs by cleavage, as in the original double-strand break repair (DSBR) model of Szostak *et al.* [5]. In this model, dHJs can be resolved in either of two equally likely orientations (Figure 1F). One orientation gives products with a single hDNA tract (upper products) and the other gives products with a tract of hDNA adjacent to a tract of gene conversion (lower products). Thus, analysis of hDNA patterns in final recombination products can be used to make inferences about the structures of intermediates that give rise to COs and NCOs.

**Figure 2:**
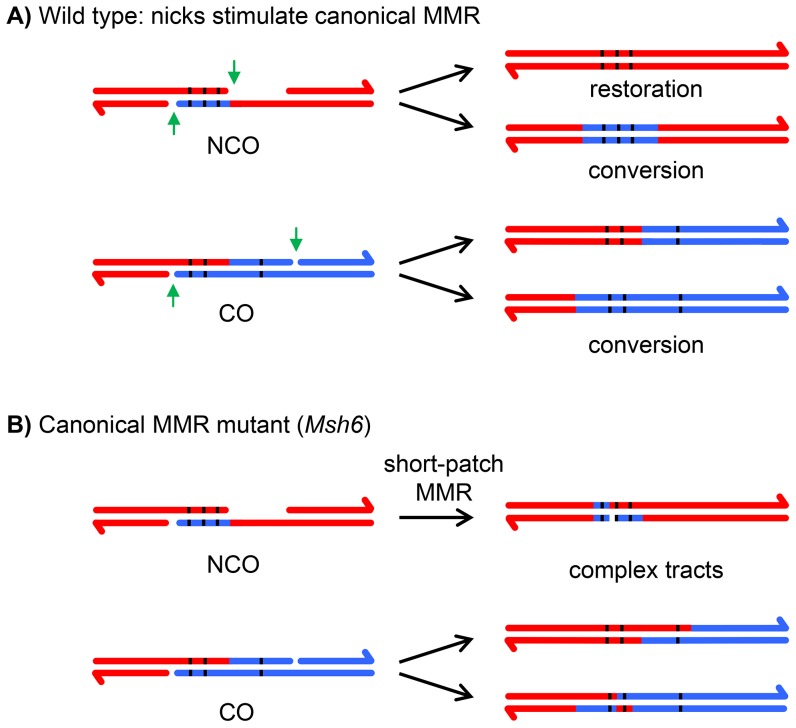
Effects of canonical and short-patch mismatch repair on hDNA correction. (**A**) In wild-type cells, canonical MMR is thought to be stimulated by the nicks (green arrows) left after repair synthesis is complete [11]. Any mismatches in the hDNA (black lines) can either be restored to the original genotype or converted; all mismatches within the hDNA are repaired in the same direction because canonical MMR repairs long tracts. In the case of crossovers, regions of gene conversion can only be detected by recovering both recombinant chromatids. If only one is recovered, as in most metazoan systems, regions of gene conversion are not detectable. (**B**) In a canonical MMR mutant, such as *Msh6*, a short-patch MMR system is able to repair mismatches. In contrast to canonical MMR, mismatches that are very close together are repaired independently of one another (or not repaired), producing complex repair tracts.

The information in hDNA is usually lost because of mismatch repair (MMR), resulting in either gene conversion or restoration of the original sequence (Figure 2A). Attempts to recover meiotic hDNA by knocking out the canonical MMR have been made in budding yeast, animals, and plants [6]–[10]. In every case, the hDNA tracts that are recovered are complex mixtures of hDNA, gene conversion, and apparent restoration (Figure 2B; we note that the term “half conversion” has been used in genetic studies to refer to retention of hDNA in the final recombination products, but we use “hDNA” to refer to regions of heteroduplex both in intermediates and in products of recombination). This complexity makes interpretations difficult because it is not possible to determine whether tracts of conversion come from synthesis-dependent processes that do not involve hDNA, such as gap repair or synthesis and dHJ resolution, or from hDNA that was repaired by a process other than the canonical MMR pathway. Similarly, apparent restoration could come from either hDNA repair or from synthesis using the sister chromatid as a template, with transitions from hDNA to restoration to conversion possibly resulting from template switching during repair.

In the canonical eukaryotic MMR pathway, recognition of mismatches and indels is dependent on heterodimers of MutS homolog (Msh) proteins, Msh2–Msh3 and Msh2–Msh6 [reviewed in 11]. *Drosophila* does not have an ortholog of Msh3 [12]; it is thought that all canonical MMR uses a heterodimer between the Msh2 ortholog (SPEL1) and MSH6. In support of this hypothesis, meiotic recombination in *Msh6* mutants resulted in hDNA tracts that were patchy, as described above (Figure 2B), suggesting that canonical MMR was eliminated [8]. It was proposed that the patchiness resulted from a short-patch MMR system that was able to repair some mismatches and small indels within the same meiotic hDNA tract independently of each other (Figure 2B).

Short-patch MMR has been reported in fungi, animals, and plants, but in most cases the proteins that execute this pathway are unknown [6], [13]–[16]. The exception is *S. pombe*, where a short-patch MMR system that depends on nucleotide excision repair (NER) operates during meiosis. This short-patch system is detected when canonical MMR is absent, and seems to repair primarily C∶C mismatches, which frequently escape canonical MMR [13]. In budding yeast, NER has recently been shown to repair mismatches containing methylated bases [17], but this pathway is not thought to be involved in repair of non-methylated mismatches [14]. In *Drosophila mei-9* mutants, a subset of meiotic hDNA tracts are able to escape both canonical and short-patch MMR [18]. MEI-9 is the *Drosophila* ortholog of *S. cerevisiae* Rad1 and mammalian XPF, the catalytic subunit of a nuclease essential for NER [19]–[22]. This suggests that NER might be involved in short-patch MMR in *Drosophila*; however, these studies were complicated by the fact that MEI-9 is also required to generate meiotic crossovers [18], [22], [23].

We now show that hDNA repair in MMR mutants in the model metazoan *Drosophila melanogaster* requires the NER protein XPC. XPC, the ortholog of *S. cerevisiae* Rad4, is involved in the DNA damage recognition step of NER [24] and has no known or suspected role in meiotic recombination. The ability to knock out both canonical and short-patch MMR allowed us to analyze uncorrected hDNA patterns, leading to novel insights into the structures of pre-CO and pre-NCO intermediates. Our findings challenge the applicability of a central paradigm of the current recombination model from budding yeast by suggesting that NCOs and COs may arise from the same intermediate.

## Results/Discussion

### Short-patch mismatch repair tracts in *Drosophila* are similar in size to NER excision tracts

**Figure 3:**
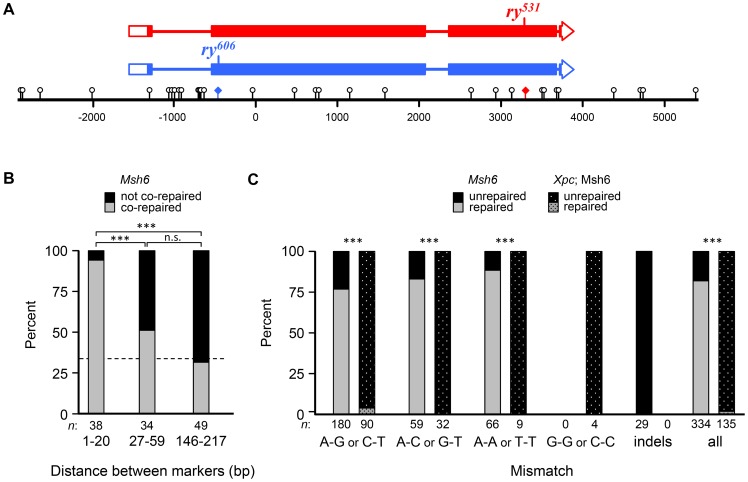
Short-patch co-repair frequencies are consistent with NER tracts. (**A**) Schematic of the *rosy* locus used to recover tracts of hDNA. Boxes represent exons and filled regions denote coding sequences. Locations of mutants are indicated on the schematics and shown on the scale bar as colored diamonds. Markers used to map hDNA tracts are shown as lollipops on the scale bar (distances in base pairs, bp). See Materials and Methods for details. (**B**) Percentage of adjacent markers that are co-repaired and not co-repaired for different distances. Bars show the percentage in each class (gray, co-repaired; black, not co-repaired) for different ranges of distance between markers. The dotted line represents the expected frequency of co-repair if adjacent markers are repaired independently. The shortest distance class is within the range of NER excision tract size. (**C**) Frequency of repair of different mismatches in *Msh6* and *Xpc*; *Msh6* mutants. Bars represent percentage of each mismatch type that were repaired (gray) or unrepaired (black). Since DSBs likely occur at different, unknown sites, we cannot tell which of two possible mismatches was in the hDNA of the intermediate (though this can be inferred for *trans* hDNA in *Xpc*; *Msh6* mutants). *Msh6* data in (B) and (C) are from Radford et al. (2007b). NCOs that had full gene conversion with no unrepaired sites were not included, since these might arise through other mechanisms (see Figure S1); however, including these tracts did not change the outcome in either case. ***, *P*<0.0001; n.s., *P*>0.05 (two-sided Fisher's exact test).

To recover hDNA tracts, we used a genetic assay to select for wild-type recombinants in the *rosy* (*ry*) gene [8],[18],[25],[26]. Briefly, when flies mutant in *ry* are exposed to dietary purine, they die as larvae. We generated females that were heteroallelic for two *ry* mutations about 4 kb apart (Figure 3A). Each *ry* allele was flanked by unique recessive markers that allowed us to determine if a recombinant was a CO or a NCO and had additional markers (single nucleotide polymorphisms (SNPs) and indels) that allowed us to map the hDNA tracts. These females were mated to males with a deletion in *ry* and allowed to lay embryos for three days. Purine was then added to the food; only wild-type recombinant larvae survived to adulthood. The presence of hDNA in the maternal *ry* allele results in mosaic larvae that have both strands of this chromosome represented in different cells or tissues. If the hDNA spans a mutant site, this results in mosaicism for *ry* activity, but *ry* is non-cell autonomous so these larvae also survive purine treatment [8]. To detect mosaicism and analyze the composition and structure of hDNA tracts, we extracted genomic DNA from the surviving recombinants and sequenced both bulk PCR product and cloned, individual molecules.

To test the hypothesis that short-patch MMR in *Drosophila* is dependent on NER, we first asked whether tract lengths are consistent with NER tracts, which extend 22–24 nucleotides 5′ and 5–6 nucleotides 3′ of the lesion being excised [27]. We analyzed previously described recombination tracts from *Msh6* mutants, which lack canonical MMR but exhibit short-patch MMR [8]. We classified each pair of adjacent markers as co-repaired (both converted or both restored) or not co-repaired (one converted and one restored or one repaired and one not repaired); pairs in which both were unrepaired were not counted. 40 of 42 (95%) pairs of markers less than 21 bp apart, and therefore within the range of NER tracts, were classified as co-repaired (Figure 3B). In contrast, when adjacent polymorphisms were further apart than the size of NER tracts, only 40 of 111 (36%) were considered co-repaired (*P*<0.0001; Figure 3B). This result supports the hypothesis that short-patch MMR in *Drosophila* is mediated by NER.

### Short-patch mismatch repair requires a key NER protein

**Figure 4:**
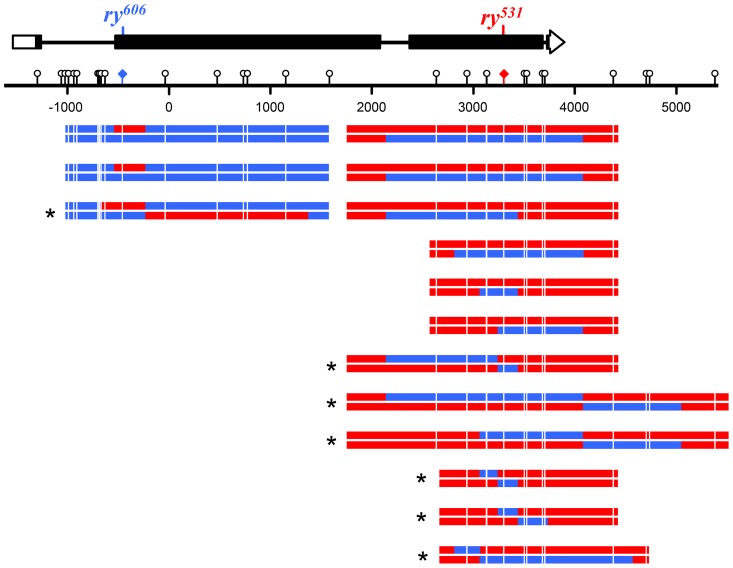
Noncrossovers from *Xpc*; *Msh6* mutants. At the top is a schematic of the *rosy* locus and the location of the mutant alleles used for purine selection (see Figure 3 and Materials and Methods). Each pair of lines below the scale represents the two strands of an independent noncrossover recombinant chromosome (red, sequence from *ry^531^* chromosome; blue, sequence from *ry^606^* chromosome). Markers used to map hDNA tracts are indicated with lollipops on the scale bar and white lines on the recombinants. Tract ends are shown as the halfway point between the last marker included in the tract and the first marker not in the tract. The two tracts at *ry^606^* that contain only a single marker were not included in the *trans/cis* analysis. Asterisks indicate tracts with *trans* hDNA.

**Figure 5:**
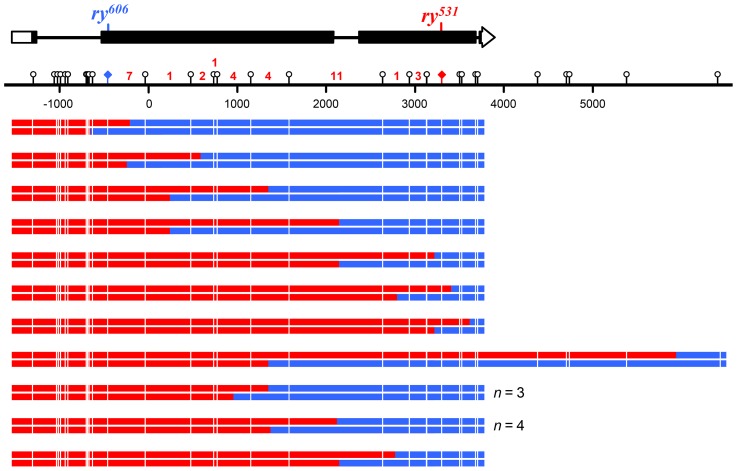
Crossovers from *Xpc*; *Msh6* mutants. At the top is a schematic of the *rosy* locus and the location of the mutant alleles used for purine selection (see Figure 3 and Materials and Methods). Each pair of lines below the scale represents the two strands of an independent crossover chromosome (red, sequence from *ry^531^* chromosome; blue, sequence from *ry^606^* chromosome). Markers used to map hDNA tracts are indicated with lollipops on the scale bar and white lines on the recombinants. Tract ends are shown as the halfway point between the last marker included in the tract and the first marker not included in the tract. All crossovers with detectable hDNA are shown. The number of crossovers without detectable hDNA that occurred within each interval are indicated in red numbers above the scale bar.

We directly tested the involvement of NER in short-patch MMR by removing XPC, a key damage recognition factor in NER [24]. Previous studies of *Xpc* (also known as *mus210*) did not report any apparent meiotic defects [28]. We screened 1.7 million larvae and recovered 66 products of meiotic recombination (50 crossovers and 16 noncrossovers) between two highly polymorphic alleles of *ry* in *Xpc*; *Msh6* double mutants. Among these recombinants we detected 32 hDNA tracts spanning a total of 136 markers (Figures 4 and 5). This does not include two noncrossovers that had tracts of full gene conversion with no hDNA (Figure S1); these likely came from residual canonical MMR due to maternal MSH6 or from an alternative recombination pathway such as double-strand gap repair, so they were excluded from further analysis.

Only two of the 136 markers (1.5%) in these tracts were repaired, both as restorations within the same noncrossover. This was the only tract that was patchy, as it also had sites with unrepaired hDNA (Figure S1). In stark contrast, *Msh6* single mutants repaired 274 of 334 hDNA markers (82%; *P*<0.0001) and 35 of 39 of hDNA tracts were patchy (90%; *P*<0.0001) [8]. Based on these data and previous work suggesting that the NER protein MEI-9 is involved in short-patch MMR [18], we conclude that short-patch MMR in *Drosophila* is indeed dependent on NER. This is the first identification of a pathway responsible for short-patch MMR in a metazoan. It is notable that, unlike in *S. pombe*, where NER-dependent short-patch MMR repairs primarily C-C mismatches [13], short-patch MMR in *Drosophila* appears to repair all types of mismatches and short insertion/deletion polymorphisms with similar efficiency (Figure 3C).

### Noncrossovers are frequently associated with *trans* hDNA

Eliminating both canonical and short-patch mismatch repair makes it possible, for the first time in a metazoan, to analyze the structures of meiotic hDNA tracts generated in the complete absence of mismatch repair, thereby providing unique insights into recombination pathways. We recovered thirteen NCOs that spanned more than one marker (Figure 4). Twelve of the thirteen NCOs occurred at the *ry^531^* locus. This is potentially due to a difference in the ability to detect NCOs at each mutation: the nearest SNP on either side of *ry^531^* is between 150–200 bp and the nearest SNP downstream of *ry^606^* is 400 bp. Additionally, the markers upstream of *ry^606^* consist of some small insertion deletion polymorphisms, while the markers around *ry^531^* are single nucleotide polymorphisms. Previous analyses at *rosy* in the *Msh6* mutant did not show the same bias in NCO location [8], [18], [26], suggesting that mutations in XPC may influence our ability to recover NCOs that span indels (Figure 3).

Surprisingly, of the thirteen NCOs with tracts that include more than one marker, only six have the *cis* hDNA arrangement predicted by SDSA; the other seven have two adjacent tracts of hDNA in the *trans* orientation (Figure 4, asterisks), an arrangement not predicted by the standard SDSA model. NCOs with *trans* hDNA were previously seen in *Msh6* mutants [8], [18]. It is possible that mutations in mismatch repair genes directly cause an increase in the frequency of the intermediate that gives rise to *trans* hDNA, possibly through mechanisms such as decreasing the frequency of heteroduplex rejection. However, the level of heterology we used in these experiments does not affect the frequency of meiotic recombination in wild-type females [29], suggesting that heteroduplex rejection is not frequent in this context. Therefore, we focus the discussion below on other sources of *trans* hDNA.

**Figure 6:**
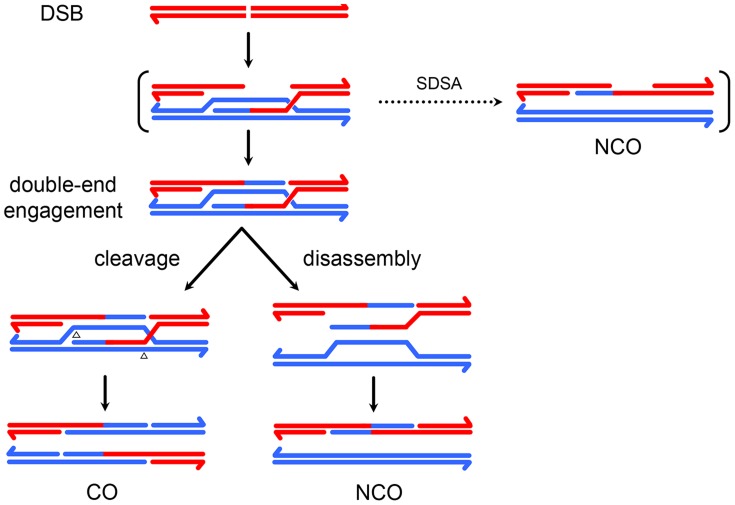
A model for meiotic recombination in *Drosophila*. At the top is a chromatid with a DSB, which enters into recombination with a chromatid on the homologous chromosome. A single-end invasion intermediate may be transient (indicated by brackets) or may give rise to some NCOs. Second-end capture and synthesis produces the two-end engagement intermediate. COs arise by nicking of this intermediate across from the existing nicks (arrowheads). NCOs arise by disassembly of the two-ended engagement intermediate by a helicase. In the version drawn here, resection is symmetric with respect to the DSB and synthesis tracts are the same length as resection tracts, resulting in nicked HJs. It is possible that resection and/or strand invasion/strand capture are asymmetric. It is also possible that synthesis does not extend all the way across the resected region, leaving a three-stranded junction instead of a nicked HJ. These possibilities, while compatible with the data, do not change the major features of the model.

A small number of NCOs with *trans* hDNA were also reported in *mei-9* mutants [8], [18]. Radford et al. [18] hypothesized that these NCOs arose from dHJ dissolution because the MEI-9 meiotic resolvase was not available to cleave the dHJs. Since canonical MMR appears to be normal in *mei-9* mutants [18], it was suggested that hDNA persisted in these NCOs because dissolution does not leave nicks that are known to stimulate MMR [11] and because short-patch MMR is defective due to the loss of the NER function of MEI-9. According to this model, if NCOs are normally produced by dHJ dissolution, then unrepaired hDNA should be frequent in NCOs from wild-type females; however, unrepaired hDNA is never detected in recombinants from wild-type females [8], [26], [30]. This argument implies that the *trans* hDNA in the NCOs we describe here arises through a process that generates products with nicks or gaps rather than through dHJ dissolution. Based on these considerations, we propose that the *trans* hDNA in *Xpc*; *Msh6* mutants comes either from either two-ended SDSA or a process we term “two-end engagement”, wherein both ends of a break engage with the same homologous chromatid and are extended by synthesis but are not ligated to produce a dHJ (see Figure 6 and discussion below). Studies of gap repair in mitotically growing yeast cells have led to the suggestion that some *trans* hDNA in NCOs comes from an intermediate with unligated HJs [31].

### Crossovers are not associated with MMR-independent gene conversion

We also analyzed crossovers generated in the absence of both canonical and short-patch MMR. In the DSBR model [5], crossovers are generated by resolution of a dHJ in either of two equally likely orientations, one of which gives products with a tract of hDNA adjacent to a tract of full conversion (Figure 1F, upper products versus lower products). Because we recover only the chromatid that goes into the oocyte, this tract of gene conversion can only be detected if there is an adjacent tract of hDNA. As drawn in Figure 1F, the model predicts that all COs have hDNA tracts, but we detected hDNA in only 16 of the 50 COs (32%). This may be a consequence of low marker density in some regions (Figure 5), since tracts that do not span a marker will not be detectable. If our ability to detect gene conversion tracts was similar to our ability to detect hDNA, then among the 16 COs with hDNA it should have been possible to detect gene conversion in five COs (32% of 16). The binomial distribution probability of recovering zero out of five is 0.04. This suggests that crossovers in *Drosophila* are not usually associated with MMR-independent gene conversion tracts.

One possible explanation for these results is that dHJ resolution is biased toward a single orientation in which nicks are made at or near the point where the 3′ end of the nascent DNA is ligated to the original resected strand (Figure 1F, open arrowheads). In yeast, a similar bias has been noted by Gilbertson and Stahl [32] and later by Jessop et al. [33]. It has been proposed for both *S. cerevisiae* meiotic recombination and DSB repair in mammalian cell lines that newly synthesized DNA provides structural asymmetry that directs cleavage to achieve this bias [34], [35]. An alternative explanation is that dHJs are un-ligated; nicking across from un-ligated HJs would also produce crossovers with an hDNA tract but no gene conversion (Figure 6). Models in which the dHJs are not ligated have been proposed to better fit the *in vitro* biochemical properties of the known structure-selective endonucleases than ligated dHJs [36].

### A unified model in which crossovers and noncrossovers come from the same two-end engagement intermediate

The high frequency of *trans* hDNA we found among NCOs, along with previous analyses of recombination in wild-type and mutant *Drosophila* [8], [18], [26], suggests that many or most NCOs may arise from an intermediate in which both resected DSB ends are engaged with the same chromatid from the homologous chromosome and are extended by synthesis. This intermediate is identical to a nicked-dHJ that we hypothesize to be a precursor to COs. Together, these results suggest the simple model illustrated in Figure 6. A central feature of this model is that both NCOs and COs come from the same two-end engagement intermediate. NCOs are produced when this intermediate is disassembled by a helicase, whereas COs are produced when it is cleaved by a structure-selective endonuclease. A two-end engagement intermediate also occurs in current models of recombination based on data from yeast (Figure 1D), but it is thought to be only a precursor to a final joint molecule with ligated HJs.

This model seems to be at odds with the argument that unrepaired *trans* hDNA in the *mei-9* mutants comes from dissolution of ligated dHJs (see discussion above and ref 8). We hypothesize that crossover formation involves protection of intermediates from helicase-catalyzed disassembly, perhaps by the mei-MCM complex [37], prior to resolution by the MEI-9 complex. In the absence of MEI-9, protection of the crossover-designated intermediate may persist until breakdown of the synaptonemal complex and recombination nodules, after which repair follows a pathway more like that in mitotic cells (similar to return-to-growth experiments in yeast). This may involve immediate disassembly or cleavage of the unligated dHJ, or ligation into a dHJ and then resolution or dissolution. MMR may occur before or after these processes. The extremely low frequency of unrepaired *trans* hDNA in the *mei-9* mutant (only 3 of 32 NCOs) suggests that we may have detected only a fraction of the events – those that were ligated and then dissolved prior to MMR; other intermediates may have been subject to MMR, either prior to or without ligation, or after resolution.

**Figure 7:**
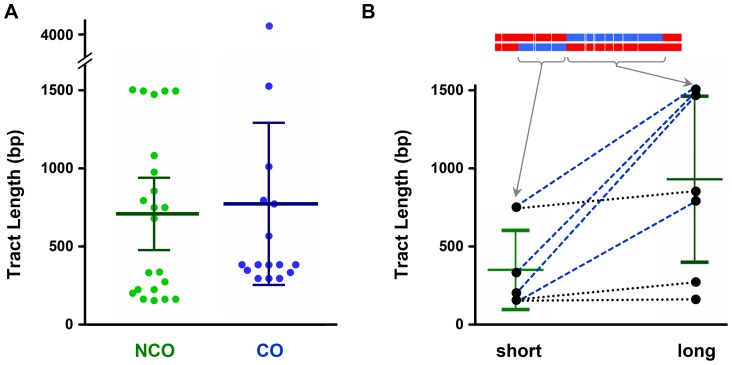
Comparison of hDNA tract lengths. (**A**) Tract lengths from noncrossovers (NCO) compared to tract lengths from crossovers (CO). Each dot represents the maximum-likelihood size of on hDNA tract (see Materials and Methods). Bars indicate mean and 95% confidence intervals. The CO mean includes one exceptionally long CO tract (4198 bp), but the difference between NCO and CO was not significant regardless of whether this tract was included (*P* = 0.7985) or excluded (*P* = 0.2901). (**B**) Relationship between lengths of the two sides of *trans* hDNA NCO tracts. The shorter side of each is graphed on the left and the longer side on the right. One example is shown at the top, with arrows pointing to the length of the short and long sides (this example is the fifth NCO from the bottom in Fig. 4, reversed so the shorter end is on the left). Short and long sides from each individual tract are connected by lines: blue dashed lines, events in which short and long sides were markedly different; black dotted lines, events in which short and long sides were similar in length. Bars show means and 95% confidence intervals. The difference is modestly significant (*P* = 0.0261).

If NCOs and COs come from the same intermediate, gene conversion tract lengths would be expected to be similar between NCOs and COs. We used a modification of TractSeq [38] to estimate lengths of hDNA tracts in NCOs and COs recovered in the absence of mismatch repair. For NCOs with *trans* hDNA, we considered each of the two halves to be an independent tract, since each is predicted to have the same origin as the single tract in NCOs without *trans* hDNA and the single tract of hDNA in COs (Figures 1 and 5). The mean length of NCO tracts was 710 bp (*n* = 22; SEM = 111 bp), in good agreement with a previous estimate of 706 bp based on analysis of purine-selected NCO gene conversions in *ry* [39]. The mean length of hDNA tracts associated with COs was 773 bp (*n* = 16; SEM = 243 bp); this is not significantly different from the NCO tract length (*P* = 0.7985) (Figure 7A).

Genetic studies in *S. cerevisiae* have found that tracts that are bi-directional, and therefore would give *trans* hDNA if unrepaired, are highly asymmetric in length with respect to the DSB [32], [33], [40], [41]. Among the seven NCOs with *trans* hDNA that we recovered, the average length of the shorter sides was 361 bp and the average of the longer sides was 939 bp (*P* = 0.0261) (Figure 7B). This suggests that asymmetry may also be a feature of recombination in *Drosophila*; however, visual inspection suggests that there may be two classes of NCO, one symmetric and one asymmetric (Figure 7B, black dotted lines versus blue dashed lines).

It difficult to make definitive conclusions about tract length differences from our data. Although whole-chromosome [42] and whole-genome [43] analyses indicate that *ry* is a typical locus with regard to recombination frequency, this frequency is nevertheless quite low. Our screening of more than a million larvae still yielded a somewhat small sample size. Also, the ability to detect hDNA or gene conversion tracts and the resolution with which they can be mapped is highly dependent on marker spacing, and the particular spacing of markers in the *ry* alleles we used may have impacted measurements for NCOs and COs differently. It should also be noted that selection for *ry^+^* recombinants should enrich for longer NCO tracts [39]. DSBs are thought to be made throughout the *ry* gene, rather than just near the 5′ end as in yeast [8], [44]. The longer a tract is, the greater the probability it will span one of the two mutant sites, which is required to generate a *ry^+^* allele that will survive purine selection. This selection does not impact COs the same way because any CO between the two *ry* mutations should be recoverable if it generates a *ry^+^* chromatid. There may be some selection against extremely long tracts, since these may cross a mutant site. In the absence of MMR, this will only matter if the wild-type allele is fully converted to the mutant allele, but we did not detect this pattern among COs (Figure 5). Further studies either at additional loci or genome-wide analyses that do not rely on selection should provide more accurate measurements of tract lengths.

### Comparison of meiotic recombination in *Drosophila* and budding yeast

In budding yeast, genetic data from several loci show that most NCO gene conversion tracts are uni-directional (*cis*), extending to only one side of the DSB [32], [33], [40], [41]. The small number of tracts in these studies that appear to be bi-directional (*trans*) have been explained as the result of multiple, closely spaced DSBs [33] or dHJ dissolution [32]. A single-end invasion intermediate has been detected in physical studies, but this is thought to be a precursor to dHJs and COs, not NCOs [4]; pre-NCO intermediates have not been detected in these assays [45], [46]. These molecular/genetic data, combined with physical analyses of recombination intermediates, have led to a model in which most NCOs arise through SDSA and there is a split into distinct NCO and CO pathways very early in repair, prior to strand invasion [2].

We found that *trans* hDNA is a common feature of NCOs in *Drosophila*: seven of the 13 NCO tracts that spanned more than a single marker had the *trans* orientation, and it is likely that at least some of the other six have *trans* hDNA that could not be discerned because one tract did not cross a marker (Figure 4). Recombination does not occur in hotspots in *Drosophila* [43], [47] so it is unlikely that any of the *trans* tracts are the result of multiple, nearby events. Rather, it seems most likely that *trans* hDNA arises when both sides of the DSB interact with a homologous template and are extended by synthesis. This can occur through any of three distinct processes. First, NCOs with *trans* hDNA may come from dHJ dissolution. Although the genetic studies discussed above found *trans* hDNA to be a rare event in budding yeast, a genome-wide analysis of meiotic recombination in mutants lacking canonical MMR found *trans* hDNA in at least 35% of NCOs [7]. The authors of this study proposed that these came from dHJ dissolution, although they could not rule out the possibility of two-ended SDSA. This implies that dissolution is a major contributor to NCOs and that a large fraction of dHJs are dissolved into NCOs, in stark disagreement with a wealth of molecular data supporting the conclusion that dHJs are resolved exclusively or primarily into COs [45]. While the contribution of dHJ dissolution to meiotic NCO production in *Saccharomyces* remains debatable, we believe, based on the arguments of Radford et al. (2007; see above discussion also), that dissolution is not the most attractive model to explain the *trans* hDNA we found in our studies.

A second possibility is two-ended SDSA, in which both ends of the DSB participate in strand exchange and synthesis. If the choice of partners is not coordinated, the two ends may engage with different homologous chromatids or one might invade the sister chromatid. Multi-chromatid intermediates have been detected in S. cerevisiae *sgs1* mutants; it is thought that Sgs1 helps disassemble such intermediates [48]. An end that has been dissociated from its original partner might then engage with a different partner, potentially giving discontinuous gene conversion tracts, as have been noted in yeast [7], [49]. Gene conversion tracts in wild-type *Drosophila* are never discontinuous [8], [18], [26], [50], [51], indicating that either multiple rounds of strand exchange, synthesis, and dissociation are not a feature of meiotic recombination or that the sister is never used as a template. Furthermore, *Drosophila* does not have homologs of any of the canonical partner choice proteins such as Red1 or Hop1, suggesting that homolog bias during strand invasion may be ensured by other mechanisms.

Two-ended SDSA might also occur such that both ends of the DSB invade the same homologous chromatid. It seems likely that steric hindrance would prevent two ends from invading the same template simultaneously, so two-ended SDSA with the same chromatid might require that one end invade and be extended by synthesis, then dissociate before the second end invades the same template. This might explain why some NCOs we analyzed did not have detectable *trans* hDNA. If the nascent sequence anneals to the second end before that second end participates in strand exchange, recombination could be completed through simple, one-ended SDSA. Conversely, if the second end does undergo strand exchange and extension then dissociation and annealing, *trans* hDNA might be produced. Two-ended SDSA occurring this way, or with one end invading each of the two chromatids on the homologous chromosome, could explain the frequent occurrence of *trans* hDNA we see.

A third mechanism that can produce *trans* hDNA involves a two-end engagement and synthesis intermediate (Figure 6). The process generating this intermediate is mechanistically distinct from two-ended SDSA because it involves 2^nd^-end capture (*i.e.*, annealing of the resected 2^nd^ end of the DSB to the D-loop strand displaced by synthesis) rather than 2^nd^-end strand exchange, followed by repair synthesis. Since we cannot physically detect recombination intermediates in *Drosophila*, we cannot distinguish between two-ended SDSA and two-end engagement; however, we favor the two-end engagement model because it also explains the absence of tracts of full gene conversion in crossover products (Figure 5).

In many organisms, including *S. cerevisiae*, meiotic DSBs are made prior to assembly of the synaptonemal complex (SC) [reviewed in 52]. Recombination is then used to promote chromosome pairing and synapsis and thus the ability to carry out multiple rounds of strand invasion into different partners might be favored via unstable short D-loops. In *Drosophila*, chromosome pairing and synapsis are achieved without recombination, and DSB formation does not occur until after chromosomes are fully synapsed [53], [54]. This likely has important consequences for how recombination proceeds. Since homologs are already intimately paired when recombination begins, the risk of strand invasion with an inappropriate template is greatly reduced, and the structure of the SC may enforce bias toward the homolog as a recombination partner. This may allow stable engagement with the homolog to be achieved early, making multiple cycles of strand exchange and dissociation unnecessary, and allowing both ends of the DSB to engage with a homologous template, as in the two-end engagement model.

### Points of crossover control

The two-end engagement model is conceptually very similar to the original DSBR model of Szostak et al. (1983) in having NCOs and COs come from a single intermediate. However, in the DSBR model, the NCO/CO outcome relies on random orientation of cleavage by resolvases, such that each dHJ resolution has an equal probability of producing NCO or CO products (Figure 1). In contrast, we propose that NCOs and COs are produced through different enzymatic activities – disassembly by a helicase and cleavage by a nuclease, respectively (Figure 6). Although current models from *S. cerevisiae* also have NCOs arising from helicase activity and COs from nuclease activity, our model differs critically in returning to a single intermediate. Consequently, the NCO/CO decision might be made and/or enforced much later than proposed in yeast – after this late intermediate is formed. In yeast, a key step in executing the CO decision involves loading of certain proteins, including the Msh4–Msh5 heterodimer, which is thought to protect recombination intermediates from disassembly by helicases [55], [56]. In contrast, Msh4–Msh5 focus dynamics suggest an earlier role, perhaps prior to the NCO/CO decision [57], [58], and *Arabidopsis msh4* mutants have defects in both COs and NCOs [59]. These observations suggest that a later NCO/CO decision, as in our model, may be widespread. This does not preclude the existence of an early decision that proceeds down an NCO pathway such as one-ended SDSA, but rather adds the possibility of introducing a second control point. In fact, studies of crossover homeostasis point to two phases of crossover designation in mice [60].

### Concluding remarks

Our analysis of *Drosophila* meiotic recombination after eliminating both canonical and short-patch MMR reveals that *trans* hDNA is frequent in NCOs and that MMR-independent gene conversion tracts are infrequent in COs. Although it is possible to fit these results to current models of meiotic recombination from yeast, doing so requires the addition of two-ended SDSA as a major contributor to NCO formation and biased crossover resolution. We favor the two-end engagement model because of its simplicity, its ability to succinctly account for all of our results, and how this model correlates with other features of *Drosophila* meiosis (*e.g.*, DSB induction after SC formation and the absence of any orthologs of the homolog bias-promoting proteins Red1, Hop1, and Dmc1). Some of these features may be specific to *Drosophila* meiotic recombination. However, reports of *trans* hDNA in the *S. cerevisiae* literature suggest that what may be a major pathway of NCO formation in *Drosophila* might also be a minor pathway of NCO formation in yeast, and the discussion above raises the possibility that a late intermediate that can be processed into CO or NCOs may also occur in mammals and in plants. *Drosophila* might provide a unique opportunity to study this pathway in more detail. Important tests of this model will include more precise determination of the frequency of *trans* hDNA in noncrossovers, measurements of hDNA tract length distributions, and assessment of whether the MEI-9 nuclease complex has a preference for unligated HJs over ligated HJs.

## Materials and Methods

### Recovery of recombination events within the *rosy* gene

Experiments were done in flies heteroallelic for two nonsense mutations in *Xpc* (also known as *mus210*) and two deletion mutations in *Msh6* [8], [28]. Thirty females of the genotype *Xpc^G1^*/*Xpc^C2^*; *P*{*GawB*}*h^1J3^ Msh6^68^ ry^531^ cv-c*/*Msh6^10^ kar ry^606^ red Sb* were crossed to 10 males of the genotype *y*/*Y, Dp(1:Y)y^+^*; *kar ry^506^ cv-c*. Purine selection was carried out on the progeny as in [8]. Briefly, adults were allowed to mate and lay eggs for three days before being removed, and then an aqueous purine solution was added to the medium. This treatment kills *ry* mutant larvae, but rare *ry^+^* recombinants survive. Previous experiments demonstrated that larvae that are mosaic due to loss of mismatch repair survive as well as fully wild-type larvae [8]. One bottle in every tray of 25 was left untreated so adult progeny could be counted to estimate the total number of larvae screened.

Previous studies of recombination at the *ry* locus demonstrated that essentially all recombinants arise during female meiosis [61]. This is evident in the observation that each treated bottle has zero or one surviving *ry^+^* adult fly. In experiments reported here, however, there were six cases of clusters of *ry^+^* progeny in a single bottle. Most or all of these appear to result from recombination between the *ry^531^* and the *TM3* balancer chromosome in the stock, prior to generating heteroallelic females. In numerous previous experiments of this type in our laboratory [8], [18], [26], [51], we have observed only a single similar case (KP Kohl and JS, unpublished). The rate may be higher in the experiments here because of simultaneous reduction in both XPC and MSH6. However, since all such events happened in one of the two stocks, it may be the presence of two balancer chromosomes (*CyO* for chromosome *2* and *TM3* for chromosome *3*) that led to an increase in recombination in the *ry* region. These events were excluded from our analysis, since they occurred in a previous meiotic or mitotic cell cycle.

### Detection and analysis of hDNA tracts

Recombinant flies were homogenized to isolate DNA. Sequences from *ry* were amplified by PCR, using primers anchored in the *ry^506^* deletion so as to amplify only the maternal, recombinant chromosome. To avoid PCR-mediated recombination, an extension time of one minute per kilobase was used and amplification was limited to 25 cycles. Bulk PCR product was sequenced to confirm whether the recombination event was a crossover or noncrossover and to map locations of gene conversion tracts and hDNA. To determine the orientation of hDNA markers on the two strands, PCR amplicons were isolated through Topo-TA cloning (Invitrogen Life Technologies) and individual colonies were sequenced. Table S1 shows the polymorphisms used in this study.

Tract lengths were estimated using a modification of TractSeq [38]. Each tract has a minimum length determined by the outmost included markers and a maximum length determined by the nearest non-included markers. TractSeq uses a truncated exponential to find the most likely length of each tract. For the variable *p*, which is the probability of extending one additional base, we used 0.99717, a value derived previously to estimate the lengths of gene conversion tracts in *ry* [39]; however, the same conclusions were reached when we varied *p* from 0.990 to 0.999, the value used by Rockmill *et al.* [38] for experiments in *S. cerevisiae*. Our modification uses the same method for tracts that include a single marker as for tracts that include multiple markers.

## Supporting Information

Figure S1 The patchy hDNA tract and tracts of full gene conversion recovered from *Xpc*; *Msh6* double mutants. The single patchy tract is shown at the top and the two fully-converted tracts below. Since each of full conversions spanned four widely-spaced SNPs, it is unlikely they are the result of residual short-patch MMR activity. A similar number of full conversions were seen in *Msh6* single mutants (4 of 35, *P* = 0.6), suggesting that these might result from residual canonical MMR. It is possible that MSH6 protein is deposited in oocytes by the heterozygous mothers and that some persists until meiosis in the daughters; however, both gene conversions shown here came from the 2^nd^ brood bottles (see Materials and Methods) and therefore from older females. Alternatively, this gene conversion might be independent of MMR and instead come from a different repair pathway. If the DSB is enlarged to a gap before repair, synthesis using the homolog will necessarily generate a tract of full gene conversion. This may explain the five cases from *Msh6* single mutants in which a single SNP was converted [8], but it seems less likely to explain the four long tracts from that study or the two long tracts illustrated above. Full conversion can also be produced by dHJ resolution (see Figure 1).
(TIFF)

Table S1 Polymorphisms between *ry^531^* and *ry^606^* used as markers to map hDNA. Positions are relative to an *Eco*RI site in the coding region (3R:8,859,890 on the genome assembly release 5.44). The *ry^606^* mutation is at −468 (bold), and the *ry^531^* is at 3312 (bold).
(DOCX)
