# Supplementary figures and images for: Eliminating Both Canonical and Short-Patch Mismatch Repair in Drosophila melanogaster Suggests a New Meiotic Recombination Model
Source: PLoS Genet. 2014 Sep 4;10(9):e1004583. doi: 10.1371/journal.pgen.1004583 (PMC4154643; doi:10.1371/journal.pgen.1004583)

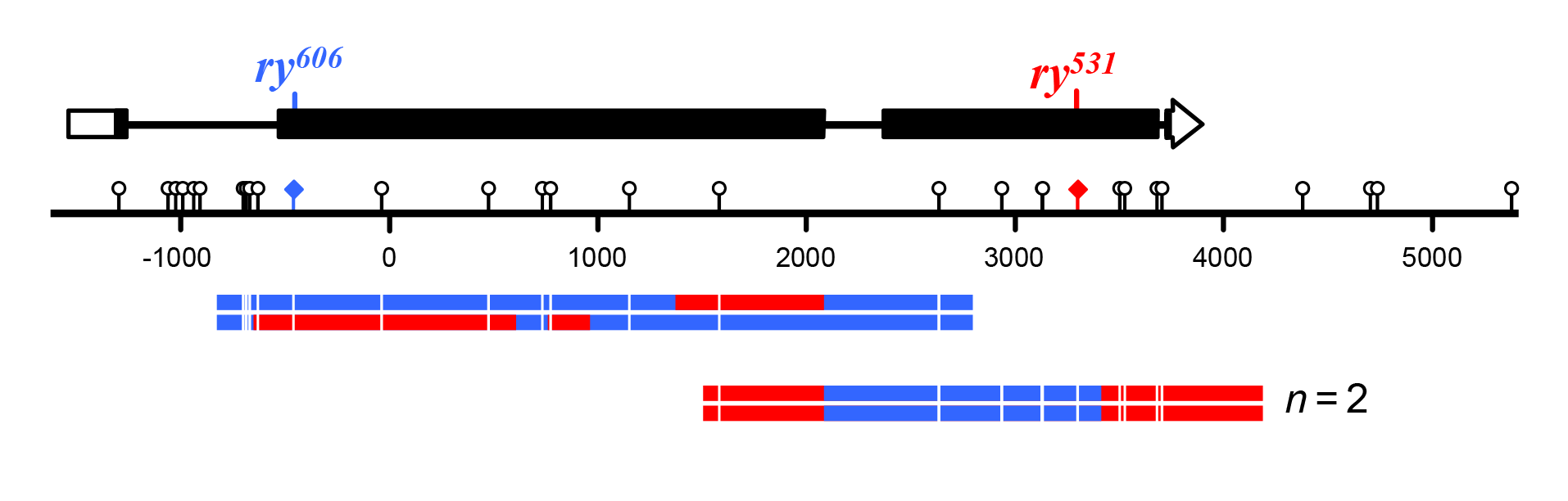

Supplement: Figure S1 — The patchy hDNA tract and tracts of full gene conversion recovered from Xpc; Msh6 double mutants. The single patchy tract is shown at the top and the two fully-converted tracts below. Since each of full conversions spanned four widely-spaced SNPs, it is unlikely they are the result of residual short-patch MMR activity. A similar number of full conversions were seen in Msh6 single mutants (4 of 35, P = 0.6), suggesting that these might result from residual canonical MMR. It is possible that MSH6 protein is deposited in oocytes by the heterozygous mothers and that some persists until meiosis in the daughters; however, both gene conversions shown here came from the 2nd brood bottles (see Materials and Methods) and therefore from older females. Alternatively, this gene conversion might be independent of MMR and instead come from a different repair pathway. If the DSB is enlarged to a gap before repair, synthesis using the homolog will necessarily generate a tract of full gene conversion. This may explain the five cases from Msh6 single mutants in which a single SNP was converted [8], but it seems less likely to explain the four long tracts from that study or the two long tracts illustrated above. Full conversion can also be produced by dHJ resolution (see Figure 1). (TIFF) [file pgen.1004583.s001.tiff]
