# Supplementary material for: Eliminating Both Canonical and Short-Patch Mismatch Repair in Drosophila melanogaster Suggests a New Meiotic Recombination Model
Source: PLoS Genet. 2014 Sep 4;10(9):e1004583. doi: 10.1371/journal.pgen.1004583 (PMC4154643; doi:10.1371/journal.pgen.1004583)
Supplement: Table S1 — Polymorphisms between ry531 and ry606 used as markers to map hDNA. Positions are relative to an EcoRI site in the coding region (3R:8,859,890 on the genome assembly release 5.44). The ry606 mutation is at −468 (bold), and the ry531 is at 3312 (bold). (DOCX) [file pgen.1004583.s002.docx]

**Table S1.** Polymorphisms between *ry^531^* and *ry^606^* used as markers to map hDNA.

| Position | *ry^531^* | *ry^606^* |
| --- | --- | --- |
| -1316 | C | T |
| -1061 | A | T |
| -1029 | T | - |
| -996 | G | T |
| -937 | A | T |
| -910 | G | A |
| -698 | A | T |
| -685 | GGGT | ---- |
| -679 | A | G |
| -668 | A | T |
| -667 | G | A |
| -636 | A | C |
| **-468** | **G** | **A** |
| -44 | T | C |
| 467 | C | T |
| 736 | A | G |
| 767 | A | G |
| 777 | A | G |
| 1153 | C | T |
| 1596 | G | T |
| 2653 | T | G |
| 2957 | T | C |
| 3148 | C | T |
| **3312** | **A** | **G** |
| 3529 | T | C |
| 3557 | G | A |
| 3703 | T | A |
| 3723 | C | A |
| 4415 | A | C |
| 4739 | C | A |
| 4777 | G | C |
| 5410 | C | G |
| 6458 | G | A |
